# Anæsthesia, or the Employment of Chloroform in Surgery and Midwifery

**Published:** 1849-11

**Authors:** 


### Part 2.—Reviews and Notices of New Works.

### ARTICLE I.

*Anæsthesia, or the Employment of Chloroform in Surgery and Midwifery.*—By Prof. J. Y. Simpson, M.D., &c.

*A Treatise on Etherization in Child-birth. Illustrated by Five Hundred and Eighty-one Cases.*---By Prof. Walter Channing, M. D. &c.

*Effects of Chloroform and of Stromg Chloric Ether, as Narcotic Agents.*—By John C. Warren, M. D., &c.

*Mere opinions and prejudgments not sufficient to settle the question of the propriety or impropriety of Anæsthetic Agents.*---Such is the caption of the first chapter of Dr. Simpson's very interesting and valuable work; and it would seem so very apparently true, that we wonder any one making pretensions to candor and fairness, would venture to dispute it. Yet no sooner was the discovery of these agents announced, than distinguished members of the profession took sides on the question, and fulminated their bulletins, without paying any respect to the facts in the case.

There is a pride of science, which is about as unreasonable and disagreeable as any other species of pride. There are yet plenty of persons who will tell the clock by algebra, and take your measure for a coat with a quadrant. Too many of our scientific men have a contempt for facts and experiments, especially when said facts and experiments contravene preconceived notions and hypotheses. They prefer incurring the risk of being called visionaries, to that of being classed amongst mere experimenters. We remember to have seen it stated that the celebrated Dr. Lardner conclusively demonstrated the impracticability of navigating the ocean by steam; and about the time he had pronounced his Q. E. D., the practical navigators had sailed a steamship from Liverpool to New York. So it was in Anæsthetics: Prof. Meigs had uttered a considerable amount of learned jargon concerning the 'culminating point of the female somatic forces,' and 'the conservative manifestation of life-force,' amounting, in the end, only to his impressions of what *might be* the result of the use of these agents; while Profs. Simpson, Channing, and others, demonstrated the *facts* in the case and thus set the question in its true light before the profession. We hope the profession will one of these days, learn that our science is dependent on a rational empiricism; (in the primitive sense of the term) and that a hypothesis is good for nothing, unless it be a natural inference from ascertained facts.

In the first chapter of this work, Prof. Simpson has given a history of the opposition to vaccination, as illustrative of the stubborn opposition which is generally offered to all great improvements. This history, besides being interesting as a professional curiosity, contains a great amount of quiet satire upon that class of philosophers who bring forward their preconceived notions as an offset to facts. We subjoin the following extracts that our readers may learn how the great discovery of Jenner was regarded by some of the eminent medical men of his day:

"Dr. Squirrell earnestly and publicly supplicated his Majesty George the Third to suppress "the destructive practice of vaccine inoculation throughout his dominions." "It ought," observed Professor Monro of Edinburg, "to be prohibited by Act of Parliament." "The College of Physicians have," exclaimed Dr. Moseley, "a duty to perform, and I trust this business will not escape them." Others, despairing of interference on the part of the King, Parliament, or Colleges, appealed to the people themselves. "It would," said Dr. Brown, "undoubtedly be downright madness to imagine *they* will condescend to encourage it." The Anti-Vaccinarian Society called upon the public "to second their efforts in supporting the cause of humanity against cow-pox injuries," and besought their aid to suppress "the cruel despotic tyranny of forcing cow-pox misery on the innocent babes of the poor—a gross violation of religion, morality, law, and humanity."

"Frightful, and even fatal consequences were boldly averred to be the direct and immediate results of vaccination.

"Deaths from cow-pox inoculation were published in the mortality bills of London. "I have," alleged Dr. Mosley, physician to the Chelsea Hospital, "seen children die of the cow-pox without losing the sense of torment even in the article of death." Dr. Rowley, physician to the St. Marylebone Infirmary, professed to publish true accounts of fifty-nine deaths from "cruel vaccination;" and added, that "when humanity reflects" on these, and (to use his own words) "a great heap of victims diseased for life, and likely to transmit to posterity, for ages, beastly chronic diseases, it is enough to freeze the soul with horror." And "it is," he exclaims, "the duty of honorable men in the medical profession to alarm mankind of the impending danger of vaccination; to warn society of the multifarious evils that await them in the form of this mild catholicon, of a sweetened potion that carries fatal poison in all its destructive particles." He elsewhere eloquently declaims against "affectionate parents being robbed of their serenity, and the minds ot tender mothers being wrung with eternal suspense," "whilst a few projectors or visionists are pursuing their deleterious projects on human victims," and perpetrating a "dangerous innovation which so many fatal facts illustrate."

"Nay, it was strenuonsly maintained and believed, that not only were various old maladies, peculiar to man, thus excited into action by the "cow-pox poison," but that different new diseases peculiar to the cow were sometimes communicated to the human constitution by vaccination. "Various beastly diseases," writes Dr. Rowley, "common to cattle, have appeared among the human species since the introduction of cow-pox mange, cow-pox abcess, cow-pox ulcer, cow-pox gangrene, cow-pox mortification, and enormous hideous swellings of the face, resembling the countenance of an ox, with eyes distorted, and eyelids forced out of their true situation; diseased joints, &c."

"This was published in 1806, eight years after Dr. Jenner's first essay on vaccination appeared. During the year subsequent to the first public announcement of his discovery, Dr. Moseley suggested the possibility of the "bestial humour" of cow-pox producing "a brutal fever exciting incongruous impressions on the brain;" and "who knows (says he) but that the human character may undergo mutations from quadrupedan sympathy, and some modern Pasiphæ may rival the fables of old?" Some, after vaccination, were actually supposed to "cough like cows," and "bellow like bulls." And one anti-vaccinist ingeniously suggested that if cow-pox were known to have existed in a family, this fact might debar the members of it from the chances of matrimony. For "it would (he remarks) be no letter of recommendation, and it would be cruel for the world to know, who had laboured under the cow-pox mange, evil, ulcer, or any other beastly disease; it might infallably injure their fortune in life, particularly in matrimonial alliances. Who would marry into any family, at the risk of their offspring having filthy beastly diseases?"

Even Cobbett, the strong-headed, and generally clear-sighted 'political rhinoceros,' as he was termed by an eminent philosopher of his day, in his advice to parents, denounces vaccination in no measured terms; strenuously recommending inoculation, which had before met with quite as fierce an opposition as cow-pox. One ingenious theologian, thought it doubtful, were all true that was claimed for vaccination whether it would prove a boon to mankind; suggesting that if men 'happen to become more healthy, it is a great chance but they would be less righteous.' It would not be wonderful if forty years hence, the profession of that day should look upon Drs. Ashwell, Meigs, Tyler Smith, and other opponents of Anæsthesia, much in the same light that we of the present day regard those who denounced vaccination.

Chap. II is taken up with the proof that ether and chloroform possess the power of annulling pain. This we think no longer needs proof, and we therefore pass it by.

In Chap. III we have an enumeration of the conditions for ensuring successful anæsthesia. These are fully detailed in the following extract:

"To produce the complete anæsthetic and soporific effects of the chloroform some conditions are necessary to be attended to. Without attending to these conditions, you will have failures. 1. The chloroform vapor must always be exhibited as rapidly and in as full strength as possible, if you desire to have its first or exhilarating stage particularly done away with, and excluded; and you effect this by giving the vapor so powerfully and speedily as to apathatize the patient at once. If you act otherwise, and give it in small or slow doses, you excite and rouse the patient in the same way as if nitrous oxide gas were exhibited. 2. In order that the patient be thus brought as speedily as possible under its full influence, the vapor should be allowed to pass into the air-tubes by both the mouth and nostrils,—and hence all compression of the nostrils, &c., is to be avoided. 2. The vapor of chloroform is about four times heavier than atmospheric air. And hence, if the patient is placed on his back during its exhibition, it will, by its mere gravitation, force itself in larger quantities into the air passages than if he were erect or seated. As to the best instrument for exhibiting the chloroform with these indications, the simple handkerchief is far preferable to every means yet adopted. It is infinitely preferable to any instrument yet seen, some of which merely exhibit it by the mouth and not the nostrils, in a small and imperfect, instead of full and complete doses; and with instruments so constructed, there is no doubt whatever that failures and exciting effects would ever and anon occur.— Besides, inhaling instruments frighten patients, whilst the handkerchief does not; and mental excitement of all kinds, from whispering and talking around the patient, is to be strictly avoided, if possible. As to the quantity required to be applied to the handkerchief, it has been stated, the average dose of a fluid drachm was generally sufficient to affect an adult; but I have latterly seldom measured the quantity used. We must judge by its *effects*, more than its quantity. The operator gathering his handkerchief into a cup like shape in his hand, should wet *freely* the bottom of the cup (so to speak,) and if the patient is not affected in a minute or so, he should add a little more. It evaporates rapidly ; and you must not wet your handkerchief, and then delay for a minute or more in applying it. It must be applied immediately. Not unfrequently, when the patient was just becoming insensible, he will withdraw his face, or forcibly push aside the handkerchief. If you *then* fail to apply it to his face and keep it there, you will be liable to leave him excited. But probably two or three inhalations more will *now* render him quite insensible. The simplest test of its full and perfect effect is some noise or stertor in the respiration. Cease it as soon as this is fully set in. But re-apply it, of course, from time to time, if it is wished to keep up its effects.

"Dr. Bennett, has spoken of the stertor or some other sympton being 'serious.' Now, this and other terms are, it is believed, calculated to excite unnecessary fear. 'Serious' was a relative and conventional term, constantly liable to be altered by increased knowledge and experience.—Twenty years ago, travelling at the rate of thirty miles an hour would have been reckoned a very serious matter. Now-a-days every one knows it was not so. The tyro looks at first upon the symptoms of an aggravated attack of hysteria as very serious. The physician of more experience knows they are not so. The stertorous breathing, the spasms, and almost convulsive symptoms, &c., sometimes produced by chloroform, may appear serious to those who have had little experience in the use of this agent. But every one who has seen much of its effects, knows that there is [not only] no danger following, but no inconvenience even left by such a show of serious symptoms."

The most singular argument as yet brought against the use of ether and chloroform, is that the alleviation of the pain of operations is unnecessary, if not improper. To us this assertion sounds like nothing short of absolute barbarity. That such an assertion should be made by men professing to possess common humanity, passes our comprehension. Of what use, we should like to know, are the tortures of the operating table, to either the patient or the surgeon? Is it a pleasure to the latter to see the writhing and listen to the shrieks of the unfortunate being before him; especially when he knows that pain may often destroy life? Do such sights and such sounds have a tendency to increase the steadiness of the operator's hand, and sustain the equanimity of his judgment? How many instances are on record of patients perishing from the mere shock of an operation? Such instances are so numerous that it is useless to quote them: and yet we are gravely assured by such men as Bransby Cooper, Magendie and Syme, that they should 'feel averse to the prevention of it,' that '*c'est peu*

*de chose de souffrir,*' and that the pains of amputation are 'nowise unbearable.' Such assertions are melancholy instances of the perverseness of human judgment, and the obstinacy with which the mind resists any truth that contravenes established opinions. Why will not such men be consistent, and refuse to administer opiates to allay the pangs of their patients? Why will they so far cross the purposes of nature as to hasten the maturation of an abcess by poultices and fomentations? Trace their line of argument to its legitimate conclusion, and it will be at once seen that it is opposed to all inventions and appliances for the comfort and convenience of mankind. In short, the opinion that the pain of a surgical operation is necessary or useful, is egregiously absurd; and opposed not only to the observations and experience of medical men in all ages, but to the common sense of mankind: and when we see men of eminence in the profession promulgate such dogmas, we cannot but conclude that much learning has made them mad.

But we have already spent more time on this objection, than its importance demands; we therefore pass to the consideration of the question: "Does Anæsthesia increase or decrease the mortality attendant on surgical operations?" On the answer to this question depends the rank which anæsthetic agents will hereafter hold in our Materia Medica. To decide this question, Dr. Simpson, with persevering and commendable industry obtained results from the principal hospitals of Europe, and has given the results of his researches, in the sixth chapter of the volume before us.—According to these tables the following results were obtained. Mr. Phillips records 1,369 cases of amputation of the thigh, leg and arm: the mortalily was about 37 per cent. In 242 cases reported from the Glasgow Hospital, the mortality was about 47 per cent. In 484 cases reported from the Parisian Hospitals, the mortality was about 49 per cent., while in 302 amputations of the same class, performed under etherization, the mortality was 23 per cent. In order to make the contrast more definite, the Professor has selected a single operation, that of amputation of the thigh; and the subjoined table exhibits the result of his researches:

*Table of the Mortality of Amputation of the Thigh, Leg, and Arm.*

| Reporter. | No. of Cases. | No. of Deaths. | Per-centage of Deaths. |
|---|---|---|---|
| Parisian Hospitals—Malgaigne, - | 484 | 273 | 57 in 100 |
| Glasgow Hospital—Lawrie, - - | 242 | 97 | 40 in 100 |
| General Collection—Phillips, - - | 1369 | 487 | 35 in 100 |
| British Hospitals—Simpson, - - | 618 | 183 | 29 in 100 |
| *Upon patients in an Etherized state,* | 302 | 71 | 23 in 100 |

We ask any candid and unprejudiced surgeon to consider this matter attentively, and then ask himself the question, not whether he is justified in administering these articles, but whether he can reconcile it to his conscience to withhold agents which would diminish the mortality resulting from surgical operations? We profess to have no opinion in the matter further than the facts before us, lead us to adopt; but with the light now before us, we should not think of undertaking a surgical operation of importance, without at least giving the patient the option of etherization.

It would be an interesting question to determine in what manner etherization lessens the mortality of surgical operations; but as this is a matter of subordinate importance, we for the present pass it by. We suppose that the opposition to the use of anæsthetics in surgery, does not at present amount to much; but their employment in midwifery still meets with determined opposition from many eminent members of our profession. The arguments brought against etherization in midwifery, are the same as those used against its employment in surgery; with some additions which we will notice. These objections are generally on moral and religious grounds. It has been seriously said, that if women were to bring forth children, without suffering the throes of travail, their love for their offspring would be diminished. Or in other words, that the strength of the maternal affection is gauged by the intensity of the parturient pains. Of course those women who have easy labors, have less love for their offspring. We merely mention this, in order that it may be seen to what extremities men may be driven in the defence of a theory.—Again it has been said that under the influence of chloroform and ether, women have been known to indulge in much improper, and even indecent conversation. We scarcely know how to reply to this objection. That any man or woman will come from the lying-in chamber, and testify to the public that the parturient woman uttered indecent or improper phrases, is something we never should have thought of. "To the pure, all things are pure;" and it would also seem that the converse of the aphorism is equally correct. We envy no man his feelings who can urge such an objection as this: and to every such person, all pure-minded and virtuous women should exclaim, '*sus apage, haud tibi spiro*.' Dr. Tyler Smith may discourse about 'reflex obstetrics,' to the end of his days, without convincing us that any person ever heard bawdy conversation from the mouth of any decent woman in labor. In all such cases, it may be set down as a settled fact, that the indecency is on the side of the *accoucheur*, and not of the *accouchee*.

As Joshua was brought forward to combat the doctrine of the diurnal revolution of the earth; so Moses has been cited as authority against abrogating the pains of labor: and as the argument has about as much force in one case as in the other, we deem it a waste of time to consider it at more length; although Dr. Simpson has, to our surprise, devoted a long chapter to it.

It has been said that the pains of labor are not sufficiently great to warrant artificial interference. A reference to the standard works on Midwiferv is a full and sufficient answer to this assertion. Any one who will take the trouble to consult the statistics recorded by Dr. Churchill, in his very valuable work on Obstetrics, cannot but be struck with the fact, that the mortality is in proportion to the suffering, and that we may look to see the number of fatal cases decrease under any method of treatment that will alleviate the intolerable agonies of preter-natural labors. It has further been urged, that etherization might have a deleterious influence on the child; but this is a mere supposition, which has been most successfully refuted by experience. The objections brought forward by Prof. Meigs, have been so triumphantly answered by Prof. Simpson,and the refutation is so generally familiar to the profession, that we deem it unnecessary to make any further allusion to it here.

Having thus briefly noticed the disadvantages alleged to be encountered in the use of etherization, it is but fair to consider the other side of the question. The first great advantage gained, is the abrogation of of pain. If pain may of itself be destructive to life, certainly any means to do away with it must be regarded as a valuable addition to the Materia Medica. That chloroform and ether accomplish this, we suppose is not doubted by any one. We however need more extended statistics before we can accurately determine the influence of etherization upon the mortality of midwifery practice. In the second place, these agents have been found eminently useful in preter-natural labors. It is, of course, much more satisfactory to practice operative midwifery when the patient is in an unconscious condition, than when she is rendered restless and unmanageable by intolerable pain.

We are told by those who have the best right to know, that etherization produces relaxation of the soft parts during labor, and thus facilitates the process. These are the main advantages gained by the use of ether and chloroform in midwifery; and although not numerous, they are very important.

It should not be disguised from the profession that there is one very serious objection against the use of anæsthetic agents, and the only one, in our opinion, which has any great force. It is alleged that death has been the result in many instances. How many deaths have been produced in this way, we do not exactly know. The largest collection of the untoward effects of chloroform, that has fallen under our notice, is that by Prof. Warren, who has given a history of 15 cases.—These cases are very clearly and fully detailed; and there are two circumstances connected with them which must arrest the attention of the most superficial observer. There are no constant pathological appearances to account for the deaths of the patients, and the operations for which the chloroform was administered were generally of a trivial character. The most frequent pathological appearance was congestion of the lungs. The brain did not evince any important lesion. In some instances the blood was found to be very fluid, and the heart soft and flaccid. It seems not unlikely that in some cases death has been occasioned by a deprivation of oxygen: the inhalers being so constructed as to exclude entirely the supply of air. In other instances a fatal result seems to have been brought about by the action of the chloroform directly upon the blood. But taking the whole subject together, the cause of the fatal result is still veiled in much obscurity, and will probably long remain so. The hypothesis of Flourens, of the successive action of poisons on the hemispheres of the brain, and the medulla oblongata, lacks exactness, and does not admit of demonstration. The error of allowing physiological speculations to influence practice, is thus finely shown up by Prof. Channing:

"Not only has the physiological objection to the use of chloroform and ether prevented Professor Meigs employing them in midwifery practice,—and will continue to do so, since it is pretty clear that this objection cannot be obviated,—but it will be perceived, that this same objection has with the professor also destroyed the authority of statistics; a science which, in matters of fact, has been of the greatest practical regard and benefit. It makes no sort of odds, that a thousand or a million cases, duly reported and authenticated, have been most successfully and happily treated by etherization. The possibility, not the *probability*,—for this is denied in the very statement of the number who have safely used it,—the *possibility* of one case proving fatal *afterwards* (not in consequence of etherization, for this cannot be determined) would seem to be regarded as a valid objection by my highly respected correspondent to his ever employing it. At least, notwithstanding the thousands of cases in which etherization has been most successfully used by others, Professor Meigs, in amount, says he has not met with one in which he has thought this agency necessary, or in which it would have been usefuly employed. The position of this distinguished professor, and the collateral support which that position, and especially his opinions in midwifery, get from the adhesion of Professors Hodge and Huston to the same, makes it a duty, in the discussion of our subject, to consider all the grounds of his not having employed the remedy of pain in labor. I do not understand, that his associates in doctrine and in practice, in this regard, have, any more than himself, employed ether or chloroform in childbirth. If they have not, is not the whole reasoning against their use strictly *a priori* in its whole nature? It is not only indifferent to, but wholly irrespective of facts, which are alike the sources and the basis of all inductive science.—Its supporters do not ask, 'What has occurred?—what has etherization done in childbirth?—how safe has it been to mother and child?' They ask what it *ought* what it *should* do, upon certain physiological principles; and which show that, as far as we can see, it ought to be, or that it is very likely to be, fatal whenever used. The friends of etherization look to the simple fact,—to what actually has happened in childbirth, after using ether or chloroform. They can learn what this truly is, both from their own observation and from that of others. They know that these remedies of pain have been widely used, and with a success which attaches to no other remedy in practical medicine. They look to the facts. They collect these; and when the time for philosophising has come, they will, with great pleasure use physiology, and all other collateral aid, in their important generalizations. While thus waiting, however, they do not reject the teachings of physiology. But in the very imperfect condition of this noble science, and more especially in that department of it which concerns the nervous system, they are willing to take the guidance of simple fact, of daily observation, in the conviction that if wisely followed, it will never lead them astray."

While there is no doubt that chloroform has in many instances caused death, there is a possibility that a greater nnmber of fatal results have been attributed to this cause, than a more close investigation of the facts will justify. It is well known that in many instances a patient has been placed upon the operating table, and before the operation has been fairly commenced, fatal syncope has supervened. If in these cases chloroform had been used, the death would have doubtless been attributed to that. Is it not possible that some of the reputed deaths from chloroform have been cases of this character?

But granting that untoward results have followed the use of anæsthetic agents; shall we therefore conclude that their use is inadmissible? By no means. Disastrous consequences have followed the use of Mercurials, Antimonials, and Opiates, even when administered by careful and skillful physicians.—It is not long since we saw a well authenticated case of death produced by a drop of Laudanum; and there are many instances of serious consequences following the use of a small portion of Calomel. Yet no one thinks of abolishing these articles from the Materia Medica. We suppose that facts warrant us in asserting, that so far, more lives have been saved than destroyed by the use of anæsthetics; and the fact that fatal results have followed their administration, should only have the effect of rendering us exceedingly careful in their use, and diligent in our enquiries to ascertain the class of cases in which they are inadmissible. It is a singular fact, that no fatal accident has as yet followed the use of ether or chloroform in obstetrical practice; at least we have not seen a well authenticated report of one.

When about to use these agents, there are some considerations which should influence us in the selection. In obstetrical practice, the chloroform possesses some advantages over the ether. Its effect is more promptly induced, and is more transient. Many practitioners will doubtless think well of it on this account. A few drops poured on a handkerchief may be administered immediately on the accession of a pain, and in this way the pain may be alleviated, without destroying the consciousness of the patient during the whole period of labor. Moreover, the quantity required to be used is small, and the odor less offensive than that of sulphuric ether; a matter of some importance in the lying-in chamber. In surgical operations, on the other hand, the ether has in some respects, a superiority over chloroform. Here plenty of time can be afforded to produce the requisite degree of insensibility before commencing the operation; and the impression produced is more lasting. Sulphuric ether has also one other advantage: fewer accidents have followed its administration. So far as we now recollect, there are on record but three cases of death produced by this article, and there is some doubt whether in these cases the result was fairly attributable to this cause.—Prof. Warren prefers chloric ether to either the chloroform or sulphuric ether. The effect of its use in his hands, has been highly satisfactory. He thinks it more prompt than the ether, and less hazardous than the chloroform. We do not know that it has been used to any great extent by any one except him; but think it worthy of a trial by the profession generally. In addition to these, various other articles have been proposed as anæsthetic agents; such as Nitrous Oxide Gas, Benzin, Aldehyde, &c., but they have been found inferior to the articles above enumerated.

Prof. Simpson made a great numbei of experiments, with the view of ascertaining whether any of the anæsthetic agents were capable of producing a local effect. He found that he could narcotize part of the body of the earth-worm, centipede, and creatures of that class; and even produce the same effect in the lower order of mammalia; but never was able to produce the same effect in man. The arm immersed for hours in the vapor of chloroform was not rendered insensible; and moreover considerable inconvenience was produced, such as heat, smarting, and a turgescence of the vessels of the part. It would, in many instances, be very desirable to produce local anæsthesia; and we yet hope to see the means devised for accomplishing it.

Besides their use in surgery and midwifery, anæsthetic agents will doubtless come to have a wide application in various diseases. We published in the last number of this

Journal, cases of Periodical Neuralgia successfully treated by chloroform. The same thing is mentioned by Prof. Simpson. We have been informed by Prof. Brainard, that he has used it in severe cases of colic, with very satisfactory results. It has been known to promptly relieve the distressing affection known as sick headache. We have seen it almost entirely arrest the chill of an ague fit. In a late number of the American Journal of the Medical Sciences, is a report of a case of Tetanus successfully treated with it: and if there is any remedy that promises to mitigate the horrors of Hydrophobia it is chloroform. We have no doubt it would effectually dispel Hysteria. In short, all acute neuralgic or spasmodic affections, if they cannot be effectually cured by this remedy, may be greatly mitigated until such time as more potent remedies can be brought to bear. In the agonies of dissolution they may be used with good results.

To sum up what has been written on the subject of anæsthetic agents, we are safe in asserting that their use has the true weight of authority in the profession. In the employment of Profs. Channing and Simpson, the use of ether and chloroform in midwifery, has been attended with results that should satisfy any reasonable man of the entire propriety of the practice. No bad result has followed in a single instance. We cannot refrain from declaring that the opinions of such men, based as they are on such an extensive experience, are worth more than all the physiological speculations promulgated by men who declare in advance that they will not be convinced; and that no amount of successful cases will satisfy them that evil results may not follow. With such persons, argument is thrown away; for when a man refuses to allow facts to influence his opinions, how is he to be convinced? We hope that the day is not far distant when such an absurd mode of philosophizing will be done away. To those who are really desirous of forming a correct opinion of this very interesting and important subject, we recommend the careful and attentive perusal of the works whose titles stand at the head of this article. They are written with a candor and impartiality that are worthy of the highest commendation. Their authors are men who have the interest of the truth at heart; and who in the midst of opposition, and even detraction, have gone calmly and quietly forward in the noble work of alleviating human suffering. Had their enterprise proved a failure, bitter would have been the denunciations pronounced against them: as it has proved eminently successful, great is the honor it should earn for them.

The work of Prof. Simpson is rather a series of hasty essays, than a regular treatise; and bears manifest indications of haste. It was written at uncertain intervals of professional leisure. He seems to have had in view the presentation of the subject alone, and has not aimed at an accurate or finished style.—Prof. Channing's work, on the contrary, is a finished and scholarly production; an honor alike to its distinguished author, the celebrated institution to which he belongs, and the press of the city which styles herself the Athens of America.

